# Filamentous sieve element proteins are able to limit phloem mass flow, but not phytoplasma spread

**DOI:** 10.1093/jxb/erx199

**Published:** 2017-06-22

**Authors:** Laura Pagliari, Sara Buoso, Simonetta Santi, Alexandra C U Furch, Marta Martini, Francesca Degola, Alberto Loschi, Aart J E van Bel, Rita Musetti

**Affiliations:** 1 Department of Agricultural, Food, Environmental and Animal Sciences, University of Udine, via delle Scienze, Udine, Italy; 2 Institute of General Botany and Plant Physiology, Friedrich-Schiller-University of Jena, Dornburgerstrasse, Jena, Germany; 3 Department of Life Sciences, University of Parma, via Usberti, Parma, Italy; 4 Department of Phytopathology and Applied Zoology, Justus Liebig University, Heinrich-Buff-Ring, Giessen, Germany

**Keywords:** *Arabidopsis thaliana*, combined microscopy, phloem mass flow, phytoplasmas, sieve element occlusion, sieve element proteins

## Abstract

In Fabaceae, dispersion of forisomes—highly ordered aggregates of sieve element proteins—in response to phytoplasma infection was proposed to limit phloem mass flow and, hence, prevent pathogen spread. In this study, the involvement of filamentous sieve element proteins in the containment of phytoplasmas was investigated in non-Fabaceae plants. Healthy and infected Arabidopsis plants lacking one or two genes related to sieve element filament formation—*AtSEOR1* (At3g01680), *AtSEOR2* (At3g01670), and *AtPP2-A1* (At4g19840)—were analysed. TEM images revealed that phytoplasma infection induces phloem protein filament formation in both the wild-type and mutant lines. This result suggests that, in contrast to previous hypotheses, sieve element filaments can be produced independently of *AtSEOR1* and *AtSEOR2* genes. Filament presence was accompanied by a compensatory overexpression of sieve element protein genes in infected mutant lines in comparison with wild-type lines. No correlation was found between phloem mass flow limitation and phytoplasma titre, which suggests that sieve element proteins are involved in defence mechanisms other than mechanical limitation of the pathogen.

## Teaser

The lack of correlation between sieve element filament formation, sieve element occlusion, and phytoplasma titre hints at an unknown role for filamentous sieve element proteins in plant defence.

## Introduction

Phytoplasmas are prokaryotic plant pathogens belonging to the class Mollicutes. They are transferred by insect vectors to the phloem, where they exercise their pathogenic influence on the plant (Lee *et al.*, 2000; Bertaccini and Duduk, 2009; Bertaccini *et al.*, 2014). Phytoplasma-associated diseases have an enormous impact on agricultural yield, being associated with disorders affecting hundreds of plant species, including many economically important crops (Namba, 2011; Bertaccini *et al.*, 2014; Valiunas *et al.*, 2015). Thus far, phytoplasma outbreak and spread can only be controlled by using insecticides against vector populations or by eradicating infected plants. Alternative strategies, such as the individuation of resistant or tolerant plants (Osler *et al.*, 2014, 2016), require a thorough notion of the physiological mechanisms underlying the interactions between plant host and phytoplasmas.

Phytoplasmas are mostly confined to the sieve elements (SEs) in phloem tissue. Although their impact on the host is of major interest for agricultural yield and basic sciences, virtually nothing is known about the basics of the interaction between phytoplasmas and SEs. Phytoplasmas greatly affect the ultrastructural organization of SEs, probably by establishing connections to acquire host-derived nutrients (Buxa *et al.*, 2015; Musetti *et al.*, 2016; Pagliari *et al.*, 2016). However, it is unclear which of the ultrastructural modifications in SEs are related to resource acquisition by phytoplasmas and which are to be regarded as protective measures of the host plant against phytoplasma spread (Musetti *et al.*, 2016; Pagliari *et al.*, 2016). In infected *Vicia faba* plants, phytoplasmas also trigger structural modifications of forisomes in SEs (Musetti *et al.*, 2013). Forisomes are spindle-shaped, giant SE protein bodies, typical of SEs in Fabaceae. They can undergo a calcium-induced dispersion that leads to their expansion and to the occlusion of the sieve pores (Knoblauch *et al.*, 2001, 2003; van Bel *et al.*, 2014). In this way, forisomes impair mass flow and may limit pathogen invasion and spread (Musetti *et al.*, 2013; van Bel, 2003; Srivastava *et al.*, 2015).

Forisome components are encoded by members of the *Sieve Element Occlusion* (*SEO*) gene family, first described in Fabaceae (Pélissier *et al.*, 2008) and subsequently found to be highly conserved among eudicotyledons (Pélissier *et al.*, 2008; Rüping *et al.*, 2010; Ernst *et al.*, 2011, 2012; Froelich *et al.*, 2011). In non-Fabaceae plants, *SEO* genes encode filamentous SE proteins. SE protein filaments and forisomes may share several structural and functional features (Rüping *et al.*, 2010; Srivastava *et al.*, 2015), but instead of the well-ordered forisome structure, filamentous SE proteins form electron-dense, variously and irregularly branched strands (Sjolund, 1997; Batailler *et al.*, 2012, Ernst *et al.*, 2012; Jekat *et al.*, 2013). In *Arabidopsis thaliana*, SE protein filament formation requires two so-called *Sieve Element Occlusion Related* (*SEOR*) genes, *AtSEOR1* (At3g01680) and *AtSEOR2* (At3g01670) (Anstead *et al.*, 2012). These two contiguous genes, together with one pseudogene (At1g67790), are located on chromosome 3 and are the sole *AtSEOR* genes identified in Arabidopsis (Rüping *et al.*, 2010; Anstead *et al.*, 2012). No functional redundancy between these genes has been detected (Anstead *et al.*, 2012). The heterodimer formation mechanism is still unclear, and AtSEOR1 and AtSEOR2 interaction seems to require one or more additional unknown protein (Anstead *et al.*, 2012; Jekat *et al.*, 2013). Even if AtSEOR1 and AtSEOR2 are the sole proteins known to be necessary for SE protein filament formation, the phloem protein 2, AtPP2-A1, encoded by the gene At4g19840, is associated with the SE protein filaments in Arabidopsis (Batailler *et al.*, 2012).

In contrast to the extensive information on forisome functioning (Knoblauch and Peters, 2004; Furch *et al.*, 2007, 2009; Srivastava *et al.*, 2015), the role of SE protein filaments in non-Fabaceae plants is still a matter of debate (Froelich *et al.*, 2011; Ernst *et al.*, 2012; Knoblauch *et al.*, 2012, 2014; Jekat *et al.*, 2013). In Arabidopsis, SE protein filament subunits are synthesized in immature SEs and assembled as large protein bodies, which disperse and relocate at the cell periphery along with SE maturation (Evert *et al.*, 1972; Ehlers *et al.*, 2000; Froelich *et al.*, 2011; Ernst *et al.*, 2012). Under biotic and abiotic stress, SE protein filaments displace from their parietal position and assemble in the SE lumen as strands or a meshwork to plug the sieve plate (Achor *et al.*, 2010; Musetti *et al.*, 2010; Froelich *et al.*, 2011; Jekat *et al.*, 2013). Yet, different approaches led to conflicting conclusions with regard to actual occluding capabilities of SE proteins in Arabidopsis (Froelich *et al.*, 2011; Ernst *et al.*, 2012; Jekat *et al.*, 2013, Knoblauch *et al.*, 2014).

Considering the fact that SE protein agglutination and plugging is a typical plant response to phytoplasma infection (Lherminier *et al.*, 2003; Gamalero *et al.*, 2010; Luna *et al.*, 2011; Musetti *et al.*, 2013), the effect of potential SE occlusion by SEOR proteins in response to phytoplasma infection in Arabidopsis was investigated here. In a multidisciplinary approach, we investigated if phytoplasma-triggered SE protein filament presence really limits the phloem flow and if this strategy can eventually limit pathogen capability to proliferate in phloem tissue. To elucidate these aspects, wild-type and *AtSEOR* or *AtPP2-A1* Arabidopsis mutant lines were used, both in healthy and in phytoplasma-infected conditions.

## Materials and methods

### Arabidopsis mutant lines

Arabidopsis mutant lines, lacking one or both *AtSEOR* genes reported to be essential for SE protein filament formation (Anstead *et al.*, 2012), were used. Seeds for the single *AtSEOR* gene knockout lines, SALK_081968C (*AtSEOR1* knockout, hereafter called *Atseor1ko*) and SALK_148614C (*AtSEOR2* knockout, hereafter called *Atseor2ko*), were obtained from the Nottingham Arabidopsis Stock Centre (NASC). Two knockout/knockdown plant lines, obtained from the Institute of Plant Biology and Biotechnology of the University of Münster (Germany), were also used. These mutants, previously described by Jekat *et al.* (2013), have the *AtSEOR1* gene knockout and the *AtSEOR2* gene knockdown (*Atseor1ko/Atseor2kd*) or the *AtSEOR1* gene knockdown and *AtSEOR2* gene knockout (*Atseor1kd/Atseor2ko*), allowing the impairment of the expression of both genes (Jekat *et al.*, 2013). To study the role of PP2 protein in filament formation, the *AtPP2-A1* gene knockout line SALK_080914C was used. All mutants were in a Columbia (Col-0) background. In Table 1, the main features of the Arabidopsis mutant lines we analysed are summarized.

**Table 1.** List of Arabidopsis mutant lines used for the experiments

Line	*Atseor1ko*	*Atseor2ko*	*Atseor1ko Atseor2kd*	*Atseor1kd Atseor2ko*	*Atpp2-a1ko*
**Gene**	At3g01680.1	At3g01670.1	At3g01680.1 At3g01670.1	At3g01680.1 At3g01670.1	At4g19840.1
**Source**	SALK_081968C (NASC)	SALK_148614C (NASC)	Jekat *et al.* (2013)	Jekat *et al.* (2013)	SALK_080914C (NASC)
**Transformation**	T-DNA insertion	T-DNA insertion	At3g01680.1 T-DNA insertion At3g01670.1 hpRNA cassettes	At3g01680.1 hpRNA cassettes At3g01670.1 T-DNA insertion	T-DNA insertion

### Plant materials and insect vectors

*Arabidopsis thaliana* plants were infected with a phytoplasma strain related to ‘*Candidatus* Phytoplasma asteris’ (‘*Ca*. P. asteris’, 16SrI-B subgroup), called Chrysanthemum yellows (CY) phytoplasma (Lee *et al.*, 2004). As extensively described by Pagliari *et al.* (2016), the fourth and fifth instars of the insect vector *Euscelidius variegatus* (Bosco *et al.*, 1997, 2007) were transferred to CY-infected daisy plants (*Chrysanthemum carinatum* Schousboe), used as the source of inoculum, for a 7 d acquisition-feeding period. Thirty days after nymph transfer, 45-day-old *A. thaliana* plants were individually exposed to three infective insects. Healthy control plants were exposed to healthy insects. At the end of the 7 d inoculation-feeding period, insects were manually removed. Both insect vectors and *A. thaliana* plants were grown at 20/22 °C, under short-day conditions (9 h light/15 h dark period).

For every analysis, fully symptomatic and healthy control *A. thaliana* plants were tested 20 d after the end of the inoculation period. For symptom observation, rosette weight measurement, ultrastructural observations, and phytoplasma titre analyses, 10 healthy and 10 infected plants from each line were used. Phloem mass flow experiments required three healthy and three infected plants per line. Finally, for gene expression, at least five healthy and five infected plants were used.

### Symptoms observation and rosette weight measurement

Symptom development was observed in 10 healthy and 10 infected plants per line, from the end of the inoculation period to the harvest for different analyses. For rosette weight, pots were saturated with water and, after 14 h, plants were harvested, cutting them at ground level. Rosette weight was immediately measured. Statistical comparisons between healthy and infected plants and among the different Arabidopsis lines were performed by the Prism 7.02 software package (GraphPad Software, La Jolla, CA, USA), using, respectively, the unpaired *t*-test and two-way ANOVA with a Dunnett’s test as post-hoc test for multiple comparisons.

### Phytoplasma molecular detection

To check phytoplasma presence in Arabidopsis, each healthy and symptomatic plant was analysed by PCR. Total genomic DNA was extracted from 100 mg of leaf tissue according to Doyle and Doyle (1990), modified by Martini *et al.* (2009). DNA concentration and purity were checked using a NanoDrop 1000 Spectrophotometer (Thermo Fisher Scientific, Wilmington, DE, USA). PCR amplifications were performed with primer pair R16F2n/R16R2 5'-GAAACGACTGCTAAGACTGG-3'/5'-TGACGGGCGGTGTGTACAAACCCCG-3' (Lee *et al.*, 1995; Gundersen and Lee, 1996), using One Advanced thermocycler (Euroclone, Celbio, Milan, Italy) in 25 µl reactions containing 2.5 mM each of the four dNTPs, 20 µM of each primer, 25 mM MgCl_2_, 5× polymerase buffer, 1 U of *Taq* polymerase (Promega, San Luis Obispo, CA, USA), and 1 µl of sample nucleic acid (~20 ng). Parameters used for 40 cycle PCRs were: denaturation at 94 °C for 1 min (2 min for the first cycle), annealing at 55 °C for 1 min, and extension at 72 °C for 2 min (8 min for the last cycle). The amplified products were analysed by electrophoresis in 1% agarose gel containing 1 µl of Gel Red™ (10 000×, Biotium, Hayward, CA, USA) per ml.

### Phloem mass flow

The phloem-mobile dye 5,6 carboxyfluorescein diacetate (CFDA) (Sigma, St Louis, MO, USA) was used to investigate phloem flow. This dye, extensively used in plant research as a marker for symplastic transport, permeates the plasma membrane in acetate form and is cleaved by cytosolic enzymes producing membrane-impermeant carboxyfluorescein (CF), which is transported by mass flow inside SEs (Knoblauch and van Bel, 1998). The phloem specificity of this dye is well documented in many different plant species, including *Vicia faba*, *Solanum lycopersicum*, *Cucurbita pepo*, *Ocimum basilicum*, *Nicotiana tabacum* (Haupt *et al.*, 2001; Hafke *et al.*, 2005; Furch *et al.*, 2010; Musetti *et al.*, 2013), and Arabidopsis (Oparka *et al.*, 1994; Froelich *et al.*, 2011; Ross-Elliot *et al.*, 2017).

As previously reported in *V. faba* (Musetti *et al.*, 2013), in healthy and infected *A. thaliana* plants a droplet of freshly prepared 1 µM CFDA solution was applied to the midrib after having removed the leaf tip. After a 1 h incubation period at room temperature, 5 mm long midrib samples were cut at a distance of ~3 cm from the CFDA application site. Sample pieces were included in 8% low melting point agarose. Sections of 100 µm thick were cut by a HM560V vibratome (Microm Microtech, Brignais, France) and collected in phosphate-buffered saline solution. Sections were examined with a Leica TCS SP2 AOBS confocal laser scanning microscope (Leica, Wetzlar, Germany) with a ×40 water immersion objective (HCX Apo 0.80), exciting CFDA with the blue argon ion laser (488 nm) and collecting emitted fluorescence from 500 nm to 545 nm. For image acquisition, instrument parameters, including pinhole diameter, laser intensity, exposure time, PMT gain, and offset, were set and held constant to avoid autofluorescence and for proper comparison between samples. As a control, unstained sections were observed at the same excitation wavelength used for the fluorochrome. For each *A. thaliana* line and condition, 10 non-serial sections from three different plants were observed.

The fluorescence level in the phloem tissue was measured and compared in healthy and diseased samples by computerized image analysis in five non-serial sections per plant, using ImageJ 1.49m software (National Institutes of Health, Bethesda, MD, USA). The grey level (in arbitrary units; 0=black, i.e. absence of signal; 255=white) was measured on the tissue in an area devoid of signal on visual inspection and assumed as background (Bacci *et al.*, 2008). The threshold was then set at twice the background, and fluorescence intensity was measured and divided for the analysed surface area. One-way ANOVA followed by a Dunnett’s test was used to determine significance, with healthy wild-type values as control. Statistical analyses of fluorescence levels were performed with the Prism 7.02 software package (GraphPad Software).

### Transmission electron microscopy

To preserve phloem tissue structure, a gentle preparation method was adopted, modifying the protocol by Ehlers *et al.* (2000), to adapt it to Arabidopsis leaves as recently reported by Pagliari *et al.* (2016). Briefly, from each plant, a 30 mm long midrib portion was excised from three fully expanded leaves of the rosette. The midrib segments were immediately submerged in a MES buffer for 2 h at room temperature. A fixation solution of 3% paraformaldehyde and 4% glutaraldehyde was used and substituted every 30 min for 6 h. Samples were rinsed for 1 h and post-fixed overnight with 2% (w/v) OsO_4_. Samples were dehydrated in a graded ethanol series and then transferred into propylene oxide. From the central part of each midrib, a 6–7 mm long piece was finally excised and embedded in Epon/Araldite epoxy resin (Electron Microscopy Sciences, Fort Washington, PA, USA). Ultrathin sections (60–70 nm in thickness) were cut using an ultramicrotome (Reichert Leica Ultracut E ultramicrotome, Leica Microsystems, Wetzlar, Germany), collected on uncoated copper grids, stained with uranyl acetate and lead citrate (Reynolds, 1963), and then observed under a PHILIPS CM 10 transmission electron microscope (FEI, Eindhoven, The Netherlands), operating at 80 kV. Five non-serial cross-sections from each sample were analysed.

### Light microscopy (LM)

To compare midrib histology and phloem development in the different Arabidopsis lines (wild-type, *AtSEOR* mutant lines, and *Atpp2-a1ko*), semi-thin sections (1 μm in thickness) of resin-embedded tissue, prepared as described above, were cut using an ultramicrotome (Reichert Leica Ultracut E ultramicrotome), stained with 1% toluidine blue, and examined using a Zeiss Axio Observer Z1 microscope (Carl Zeiss GmbH, Munich, Germany). Five samples per line and condition were examined. From each sample, at least five non-serial cross-sections were observed. Phloem thickness was measured in three different non-serial cross-sections from five healthy and five infected samples per line. Three different measuring points were chosen randomly in each cross-section. Statistical analyses were performed with the Prism 7.02 software package (GraphPad Software) using the Mann–Whitney test and two-way ANOVA, and a Dunnett’s test as post-hoc test for multiple comparisons.

### RNA extraction and gene expression analyses

Total RNA was extracted from ~1 g of leaves, ground in liquid nitrogen into fine powder, and homogenized in 5 ml of lysis buffer (MacKenzie *et al.*, 1997). Homogenate (1.5 ml) was collected and centrifuged for 6 min at 12000 rpm. A 1 ml aliquot of supernatant was mixed with 100 μl of 20% (v/v) *N*-lauroylsarcosine (Sigma-Aldrich) buffer and incubated for 15 min at 70 °C. Samples were then transferred to a QIAshredder spin column (lilac) and RNA purified with an RNeasy Plant Mini Kit (Qiagen GmbH, Hilden, Germany) according to the manufacturer’s instructions. Extracted RNAs were DNase treated and reverse-transcribed into cDNA with the QuantiTectReverse Transcription Kit (Qiagen GmbH) following the manufacturer’s instructions. The expression of *AtSEOR1*, *AtSEOR2*, and *AtPP2-A1* genes was analysed in healthy and infected plants by real-time experiments performed on a CFX96 instrument (Bio-Rad Laboratories, Richmond, CA, USA). The reference gene was individuated comparing *UBC9* (ubiquitin-conjugating enzyme 9), *TIP41* (TIP41-like family protein), *SAND* (SAND family protein), and *UBQ10* (polyubiquitin 10) gene expression (Table 2). The gene stability measures (M values) were calculated according to the geNorm program (Vandesompele *et al.*, 2002) (Table 2). The *UBC9* gene was found to be the most stably expressed gene and so the most suitable as reference gene.

**Table 2.** List of primers and accession number of sequences used for housekeeping gene individuation

Gene	Forward primer 5′–3′	Reverse primer 5′–3′	nM	M value	NCBI accession no.
*UBC9*	TCACAATTTCCAAGGTGCTGC	CGAGCAGTGGACTCGTACTT	300	0.43	NM_179131.3^*a*^ NM_118934.3^*a*^
*TIP41*	CCTCTTGCGATTTTGGCTGAG	ACGAAGAACAGTTGGTGCCT	400	0.52	NM_119592.5
*SAND*	AGATCAATCGCGGAAGGTGG	TATGTCGGGACCAGGTGAGT	400	0.74	NM_128399.4
*UBQ10*	CGTCTTCGTGGTGGTTTCTAA	ACAAGGCCCCAAAACACAAAC	300	0.59	NM_178968.5^*a*^ NM_001084884.5^*a*^ NM_001340546.1^*a*^ NM_116771.5^*a*^ NM_202787.4^*a*^ NM_001340547.1^*a*^ NM_178969.6^*a*^ NM_178970.5^*a*^

^*a*^This primer pair amplifies every gene transcript variant.

SsoFast EvaGreen Supermix (Bio-Rad Laboratories Inc., Hercules, CA, USA), cDNA obtained from 5 ng of RNA, and specific primers were used in a total volume of 10 μl for *AtSEOR1* and *AtPP2-A1* genes. *AtSEOR2* gene expression analyses were carried out with cDNA from 10 ng of RNA in a total volume of 20 μl. Every reaction was performed at 95 °C for 3 min, 40 cycles of 95 °C for 5 s, and 58 °C for 5 s, followed by a melting curve analysis from 65 °C to 95 °C to check primer specificity. Primers were designed using Primer3 software (http://bioinfo.ut.ee/primer3-0.4.0/primer3/) and primer specificity evaluated with the BLASTN (Nucleotide Basic Local Alignment Search Tool) algorithm (Altschul *et al.*, 1997). Primer pair efficiency (E) was evaluated as described by Pfaffl (2001) on the standard curves of different dilutions of pooled cDNA. Gene and primer sequences for expression analysis are reported in Table 3. A mean normalized expression (MNE) for each gene of interest (Muller *et al.*, 2002) was calculated by normalizing its mean expression level to the level of the *UBC9* gene. Three technical repeats and at least five individuals concurred with gene MNE determination.

**Table 3.** List of primers and accession number of sequences used in real-time PCRs

Gene	Forward primer 5′–3′	Reverse primer 5′–3′	nM	E (%)	NCBI accession n.
*AtSEOR1*	ACCATCTCGCTGAGACCTTGAGG (Anstead *et al.*, 2012)	GGCCGTGAGAATCTTCATGTTATCA (Anstead *et al.*, 2012)	500	97.8	NM_111034.3
*AtSEOR2*	TTCAAAGAGACGCGTCGGG	GCTGCCATGCTTCTGTGTAG	500	104.0	NM_111033.3
*AtPP2-A1*	GGTGGACGAGAGAAACAGCA	GCTTCCACATTCTCGTTTGGT	400	96.3	NM_118104.5
*UBC9*	TCACAATTTCCAAGGTGCTGC	CGAGCAGTGGACTCGTACTT	300	97.1	NM_179131.3^*a*^ NM_118934.3^*a*^

^*a*^This primer pair amplifies both gene transcript variants.

Statistical analyses of gene expression levels were performed with the Prism 7.02 software package (GraphPad Software) using an unpaired *t*-test and two-way ANOVA, and a Dunnett’s test as post-hoc test for multiple comparisons.

### Phytoplasma quantification

Total genomic DNA was extracted from 1 g of leaf tissue as described above for phytoplasma molecular detection. The ribosomal protein gene *rplV* (*rpl22*) was chosen as target for the amplification of CY phytoplasma DNA using the primer pairs rp(I-B)F2/rp(I-B)R2 5'-CGTTTGGGTGGTGCTGAAAT-3'/ 5'-GAGGGCGTCTGTTAGGAGTG-3' (this study; Lee *et al.*, 2003) and producing an amplicon of 232 bp. To quantify CY phytoplasma DNA, a 1260 bp ribosomal protein fragment from CY phytoplasma, amplified with primer pair rpF1C/rp(I)R1A (Martini *et al.*, 2007), was cloned in pGem^®^T-Easy vector (Promega). Plasmid DNA was first quantified by using a Qubit^®^ 2.0 Fluorometer (Invitrogen, Carlsbad, CA, USA); then a standard curve was established by 10-fold serial dilutions of plasmid DNA corresponding to ~10^9^ to 10^1^ target genomes. Standards and 1 ng of each DNA sample (run in three replicates) were added to a mixture containing 0.3 µM of each primer and 7.5 µl of 2× SsoFast EvaGreen Supermix (Bio-Rad Laboratories) in a 15 µl total volume. Cycling conditions were as follows: initial denaturation at 98 °C for 2 min; 44 cycles of 5 s at 98 °C and 5 s at 60 °C; and a final extension at 95 °C for 1 min. A melting curve analysis (ramp from 65 °C to 95 °C at 0.5 °C s^–1^) was programmed at the end of the cycling reaction to evaluate the purity of the amplification product. CY phytoplasma concentration was expressed as the number of CY phytoplasma genome units (GUs) per mg of leaf sample to normalize the data.

The comparisons of phytoplasma population size were performed analysing the quantification results from three technical repeats of 10 plants per line with the Prism 7.02 software package (GraphPad Software), using one-way ANOVA, and a Dunnett’s test as post-hoc test for multiple comparisons, with the wild-type line as control.

## Results

### Phytoplasma detection

Phytoplasma detection was performed in healthy and symptomatic plants. Direct PCR analysis allowed the amplification of a 1250 bp fragment, confirming the presence of phytoplasmas only in the symptomatic plants (not shown).

### Phenotypes of healthy and fully symptomatic Arabidopsis plants

**Fig. 1.**
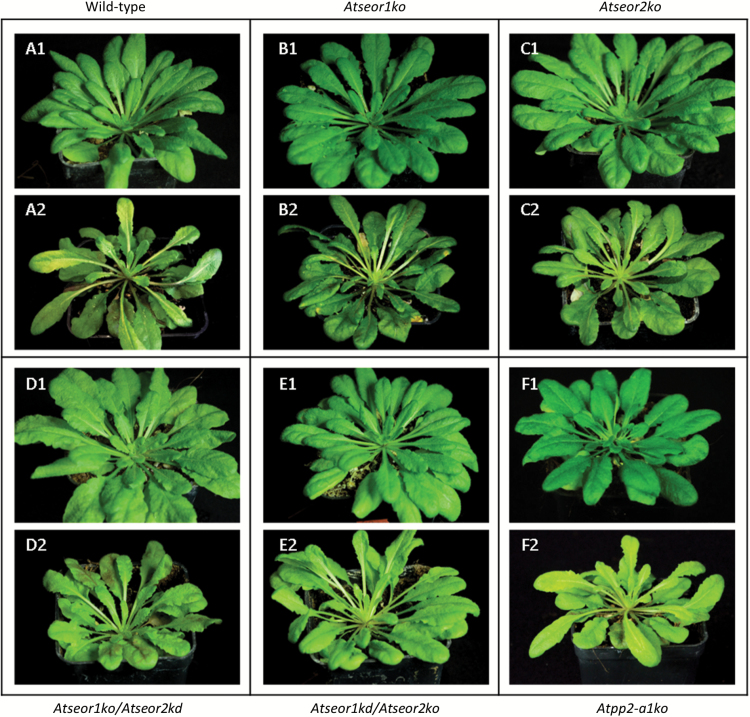
Phenotypes of representative heathy and fully symptomatic plants at 70 d. Different *A. thaliana* lines were indicated as follows: (A) wild-type, (B) *Atseor1ko*, (C) *Atseor2ko*, (D) *Atseor1ko/Atseor2kd*, (E) *Atseor1kd/Atseor2ko*, and (F) *Atpp2-a1ko*. Phytoplasma infection altered the phenotype of *A. thaliana*, producing classic phytoplasma disease symptoms. Healthy plants (1) showed regular growth, while infected plants (2) were characterized by reduced growth and shorter, yellowish leaves, with a thick main vein. No obvious difference was recorded among the different plant lines. For each condition, 10 different plants were analysed.

**Fig. 2.**
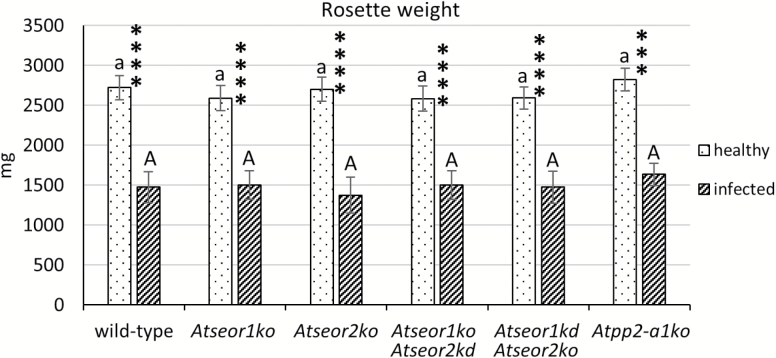
Fresh rosette weight of healthy and infected wild-type and mutant *A. thaliana* lines. The results are expressed as the mean ±SD. Infected plants were characterized by reduced growth in every line considered. No difference between wild-type and mutant lines was recorded, or in healthy or infected plants. For each condition, 10 different plants were analysed. Differences between healthy and infected conditions in each line were calculated using unpaired *t*-test. Family-wise significance and confidence level: 0.05. ****P<0.0001. For comparisons among the different lines, two-way ANOVA followed by a Dunnett’s test was used, with wild-type values as control. Family-wise significance and confidence level: 0.05. A, a: no significant difference from wild-type values.

Ten healthy and 10 infected plants from each line (the wild type, *AtSEOR* mutant lines, and *Atpp2-a1ko* lines) were observed and weighted. Healthy plants of all lines grew at similar rates (Figs 1, 2). Within 20 d from inoculation, common phytoplasma disease symptoms appeared in every plant exposed to three infective insects. Infected plants showed yellowing, reduced growth, and general stunting (Fig. 1). Leaves having emerged after phytoplasma inoculation were shorter, with a thick main vein and a smaller petiolar area. Wild-type and mutant lines did not differ in symptom development or plant phenotype due to infection (Fig. 1). A decrease of ~40% in growth was observed in all infected plant lines compared with the healthy ones, but no differences between the plant lines were observed (Fig. 2).

### Midrib morphology

**Fig. 3.**
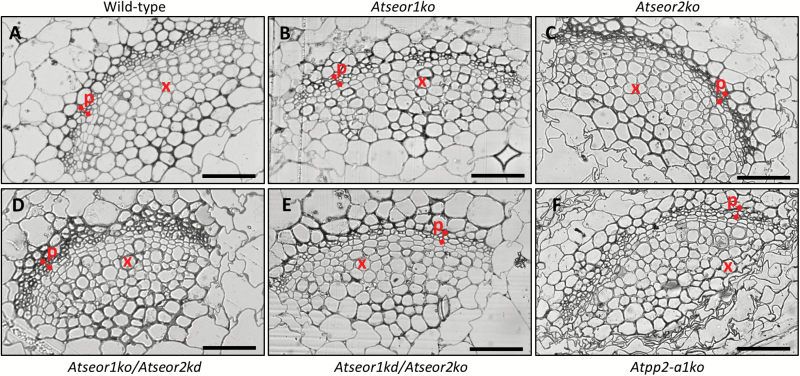
Semi-thin sections of midribs from healthy wild-type and mutant *A. thaliana* lines. Different *A. thaliana* lines were indicated as follows: (A) wild-type, (B) *Atseor1ko*, (C) *Atseor2ko*, (D) *Atseor1ko/Atseor2kd*, (E) *Atseor1kd/Atseor2ko*, and (F) *Atpp2-a1ko*. In healthy plants, vascular bundles had a regular pattern and no cell alteration. No obvious difference was recorded among the different plant lines. For each condition, at least five non-serial cross-sections from five samples were observed. p, phloem; x, xylem. Scale bars: 50 µm. (This figure is available in colour at *JXB* online.)

**Fig. 4.**
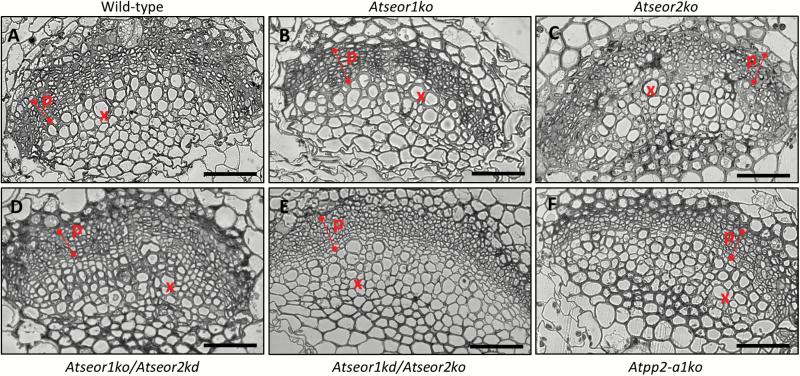
Semi-thin sections of midribs from infected wild-type and mutant *A. thaliana* lines. Different *A. thaliana* lines were indicated as follows: (A) wild-type, (B) *Atseor1ko*, (C) *Atseor2ko*, (D) *Atseor1ko/Atseor2kd*, (E) *Atseor1kd/Atseor2ko*, and (F) *Atpp2-a1ko*. In infected leaf tissues, massive production of new phloem components caused phloem hyperplasia. No obvious difference was recorded among the different plant lines. For each condition, at least five non-serial cross-sections from five samples were observed. p, phloem; x, xylem. Scale bars: 50 µm. (This figure is available in colour at *JXB* online.)

**Fig. 5.**
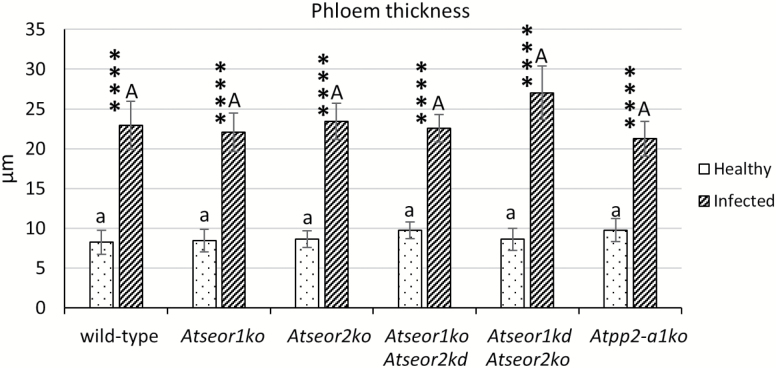
Midrib phloem thickness in healthy and infected wild-type and mutant *A. thaliana* lines. Following infection, a strong increase in phloem size occurred, due to phloem hyperplasia. Both among healthy plants and among infected plants, no significant difference was detected. The phloem thickness was measured in three different randomly selected measuring points in three different non-serial cross-sections from five healthy and five infected samples per line. The results are expressed as the mean ±SD. For each condition, five non-serial sections from five different plants were analysed. Differences between healthy and infected conditions in each line were calculated using Mann–Whitney test. Family-wise significance and confidence level: 0.05. ****P<0.0001. For comparisons among the different lines, two-way ANOVA followed by a Dunnett’s test was used, with wild-type values as control. Family-wise significance and confidence level: 0.05. A, a: no significant difference from wild-type values.

The midrib morphology in the different Arabidopsis lines was observed in at least five non-serial cross-sections from five samples per line and condition. Semi-thin sections and LM observation of midribs from healthy *A. thaliana* plants disclosed a regular collateral pattern and no cell alteration in the vascular bundles (Fig. 3). No differences between the mutant and wild-type lines were detected (Fig. 3). Phytoplasma-infected midribs were characterized by a hyperactivity of the cambial tissue, resulting in a massive production of phloem components, leading to phloem hyperplasia (Fig. 4). The increased phloem thickness was comparable in the different lines (Fig. 4), as confirmed by the measurement of the phloem thickness in healthy and infected plants (Fig. 5). Measurements were performed at three different points in three different non-serial cross-sections from five samples per line (Fig. 5).

### Phloem mass flow analysis with CFDA dye

**Fig. 6.**
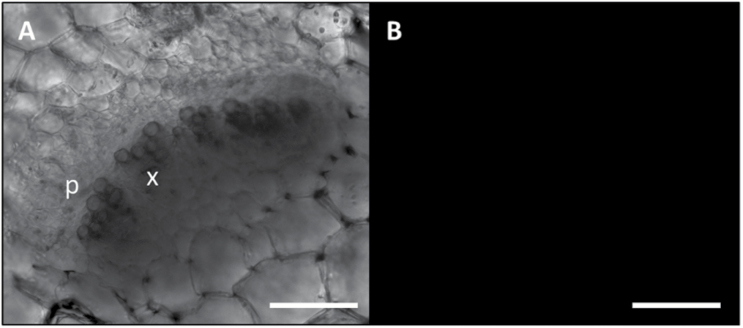
Unstained vibratome section of fresh tissue as staining control. CLSM images of unstained section from healthy wild-type *A. thaliana* midribs were visualized in bright field (A) and at the same wavelength used for the fluorochrome (excitation, 488 nm; emission, 500–545 nm) (B). No autofluorescence signal was detected. p, phloem; x, xylem. Scale bars: 75 µm.

**Fig. 7.**
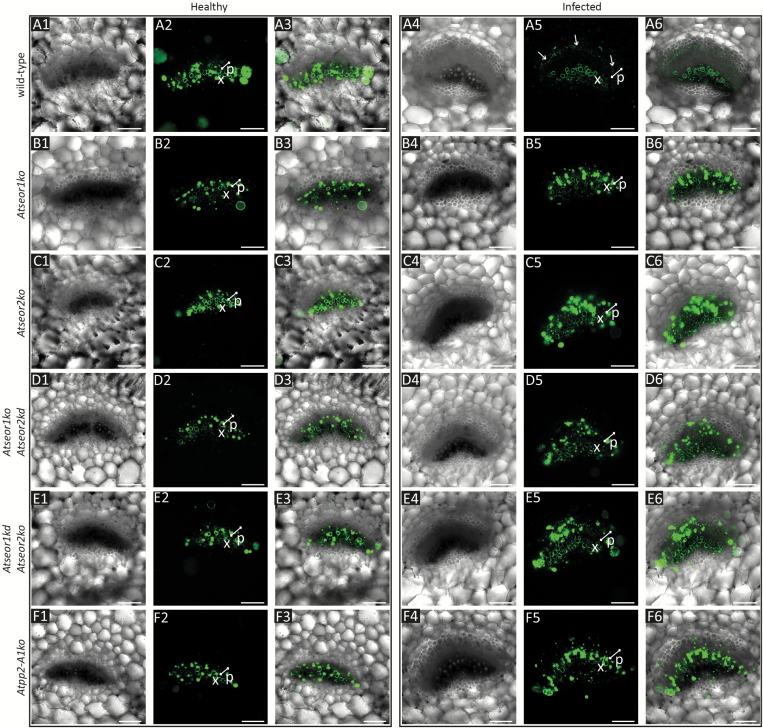
CF fluorescence signal in vibratome sections of fresh tissue of healthy and infected wild-type and mutant *A. thaliana* lines. The fluorescence of CF in the sieve elements of wild-type and mutant plants was observed at cross-sections, ~3 cm away from the dye application site. In healthy plants (1–3), CF emitted a strong signal corresponding to the phloem area in every line considered. In infected plants (4–6), the phloem tissue was larger than that observed in healthy plants (phloem size is indicated by a white bar), due to phloem hyperplasia. Whereas in wild-type plants (A) phytoplasma infection led to a modest CF signal, mutant lines (B–F) showed a high fluorescence level and dye accumulation in phloem cells. For each line and condition, 10 non-serial sections from three different plants were observed. (A) Wild-type, (B) *Atseor1ko*, (C) *Atseor2ko*, (D) *Atseor1ko/Atseor2kd*, (E) *Atseor1kd/Atseor2ko*, and (F) *Atpp2-a1ko*. p, phloem; x, xylem. Scale bars: 75 µm.

**Fig. 8.**
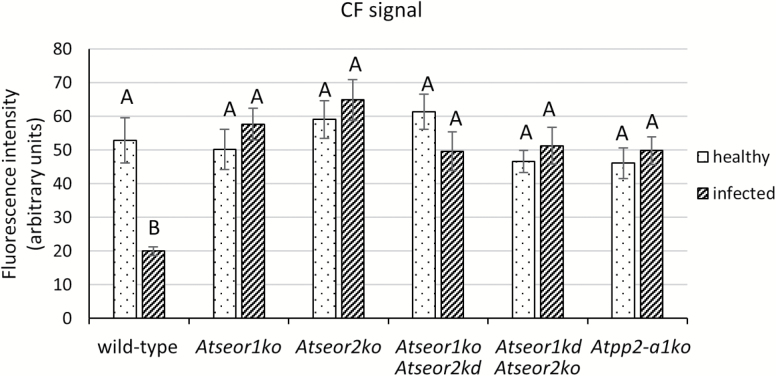
CF fluorescence quantification of fresh tissue of healthy and infected wild-type and mutant *A. thaliana* lines. The fluorescence level in the phloem tissue was measured and divided for the analysed surface area. The results are expressed as the mean ±SD. Whereas in healthy plants no significant difference was detected, in infected plants the wild-type line showed a dramatic decrease in CF signal. For each condition, five non-serial sections from five different plants were analysed. One-way ANOVA followed by a Dunnett’s test was used to determine significance, with healthy wild-type values as control. Family-wise significance and confidence level: 0.05. Different letters indicate different significance levels: A, no significant difference from wild-type values; B, *P*<0.01.

To examine alterations of phloem mass flow due to infection in diverse SE protein mutants, the location and fluorescence intensity of CFDA were observed in main vein leaf cross-sections under confocal laser scanning microscopy (CLSM). For each *A. thaliana* line, 10 non-serial sections from three different plants were observed at ~3 cm distance from the site of CFDA application. Unstained sections were observed at the same excitation wavelength used for the fluorochrome, and no fluorescent signals were detected (Fig. 6). In healthy plants of each line investigated, the phloem-mobile CF emitted strong signals from the phloem area (Fig. 7, 1–3). Colourless CFDA must have entered the SE and have been cleaved to produce CF by the activity of an intracellular esterase (Knoblauch and van Bel, 1998). All living cells at the uptake surface absorbed and cleaved CFDA, but the non-membrane-permeant CF moved away from the site of application only in the SEs, passing through the sieve pores, driven by mass flow, as seen in many different plant species, including Arabidopsis (Oparka *et al.*, 1994; Haupt *et al.*, 2001; Hafke *et al.*, 2005; Froelich *et al.*, 2011; Musetti *et al.*, 2013; Ross-Elliot *et al.*, 2017). Along the pathway, CF moves into the companion cells, where it accumulates in the vacuoles (Knoblauch and van Bel, 1998). No significant differences between the aggregate fluorescence levels in the phloem of healthy plants of the various lines were detected (Fig. 7, 1–3). Due to the high hydration and the softness of Arabidopsis tissue, cross-sectioning by vibratome caused some CF contamination. This is probably due to leakage of CF from cut sieve tubes into the apoplast, where CF adhered to intracellular components of other cut vascular cells and xylem vessel walls (Fig. 7, 1–3). In infected plants, the phloem areas were larger than in healthy plants due to phloem hyperplasia, the excessive production of phloem tissue due to procambial or cambial hyperactivity following phytoplasma infection. Infected wild-type plants showed lower CF signals in the phloem than healthy wild-type plants (Fig. 7A). In contrast, the CF emission from the phloem area in infected mutant lines was comparable with that in healthy plants (Fig. 7B–F). The interpretation of the observations was corroborated by a fluorescence quantification analysis that revealed a strong reduction of fluorescence in the infected wild-type line in comparison with all other specimens (Fig. 8).

### SE changes in ultrastructural organization following infection

**Fig. 9.**
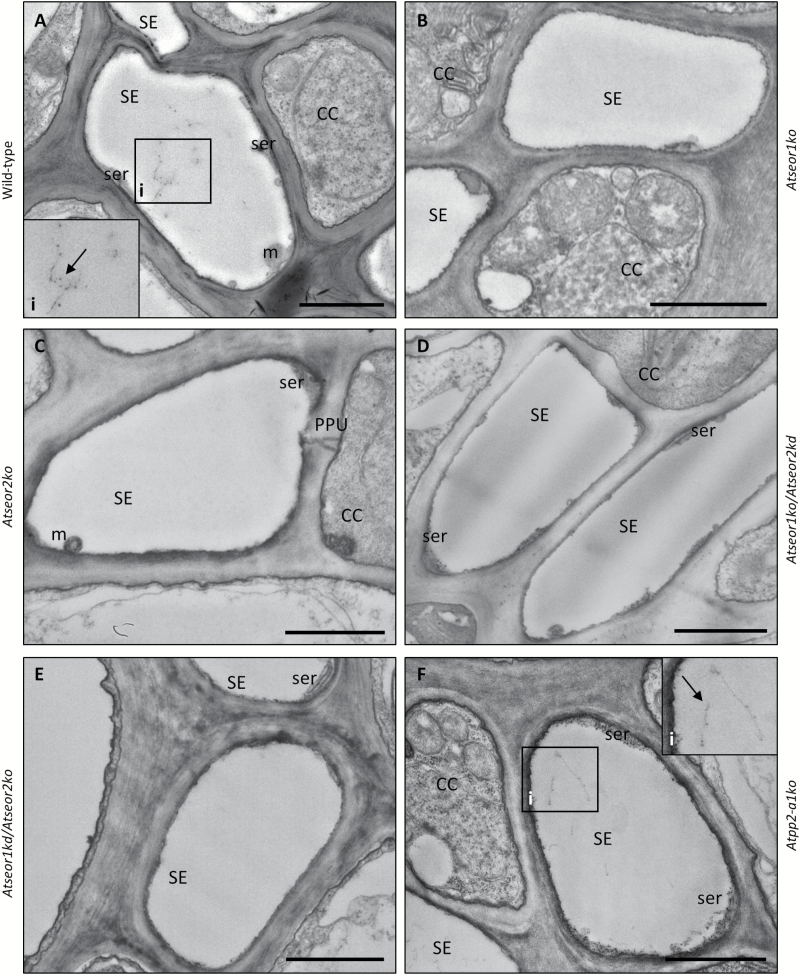
TEM micrographs of sieve elements in different healthy *A. thaliana* lines. Different *A. thaliana* lines were indicated as follows: (A) wild-type, (B) *Atseor1ko*, (C) *Atseor2ko*, (D) *Atseor1ko/Atseor2kd*, (E) *Atseor1kd/Atseor2ko*, and (F) *Atpp2-a1ko*. Healthy samples presented unaltered phloem tissue, as was also the case in transformed plants (B–F). In wild-type (A) and in *Atpp2-a1ko* plants (F), some protein filaments were observed (arrows, insets). On the other hand, lumina of mutants lacking one or both *AtSEOR* genes (B–E) appeared empty, without any sign of filament formation. Five non-serial sections from 10 different plants were analysed for each condition. CC, companion cell; m, mitochondrion; PPU, pore plasmodesma unit; SE, sieve element; ser, sieve element reticulum. Scale bars: 1 µm.

**Fig. 10.**
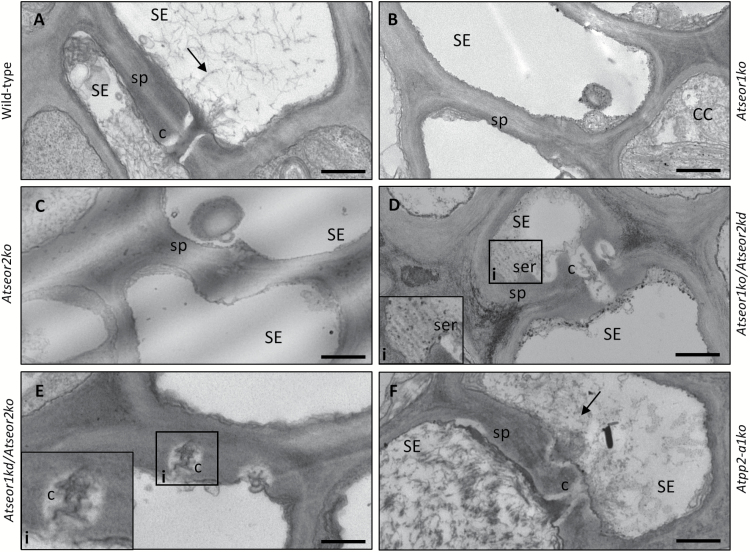
TEM micrographs of sieve plates in different healthy *A. thaliana* lines. Different *A. thaliana* lines were indicated as follows: (A) wild-type, (B) *Atseor1ko*, (C) *Atseor2ko*, (D) *Atseor1ko/Atseor2kd*, (E) *Atseor1kd/Atseor2ko*, and (F) *Atpp2-a1ko*. Sieve plates were identified at the connection between two SEs, by the presence of sieve pores (black arrowheads). Observations of the cross-sections at the sieve plate level showed SE lumina characterized by the presence of SE protein filaments (arrows) in wild-type (A) and *Atpp2-a1ko* plants (F). In mutant lines lacking *AtSEOR* genes, SE lumina appeared devoid of filaments (B–E). In *Atseor1kd/Atseor2ko* (D), the lumen of one SE was occupied by sieve element reticulum, whose stacks were visible in the inset (white arrowheads). Five non-serial sections from 10 different plants were analysed for each condition. c, callose; CC, companion cell; SE, sieve element; ser, sieve-element reticulum; SP, sieve plate. Scale bars: 500 nm.

To visualize changes in SE ultrastructure due to mutation or pathogen presence, and eventually correlate them with phloem impairment, phloem tissue was examined under TEM. For each condition, five non-serial sections from 10 different plants of each plant line were analysed. Observations of healthy samples showed a well-preserved sieve tube ultrastructure, with a regular shape and no signs of necrosis or subcellular aberrations, both in wild-type plants and in mutants (Fig. 9). In the latter lines, the SE plasma membrane had a regular profile, appressed to the cell wall. Mitochondria and thin stacks of sieve endoplasmic reticulum (SER) adhered to the plasma membrane. In wild-type plants, SE protein filaments were seldom observed and only tiny filaments were detected (Fig. 9A). In mutant plants lacking one or both *AtSEOR* genes, SE lumina were fully devoid of filaments (Fig. 9B–E). In contrast, SE lumina of *Atpp2-a1ko* plants showed some filaments that did not appear to differ from wild-type protein filaments (Fig. 9F). The absence of SE protein filaments in mutants lacking one or both *AtSEOR* genes was confirmed in the sieve plate region in the different lines. Sieve plates were characterized by a significant presence of protein filaments in wild-type and *Atpp2-a1ko* plants (Fig. 10A, F), while they were totally free from filaments in the other mutant lines (Fig. 10B–E).

**Fig. 11.**
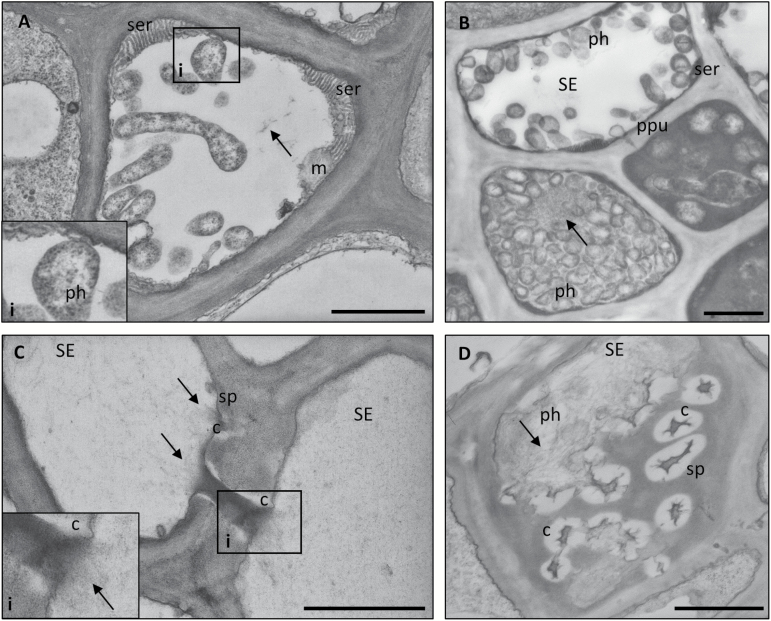
Phytoplasma distribution and ultrastructural responses in sieve tubes of wild-type *A. thaliana* plants. Phytoplasmas presented their typical pleomorphic profile, delimited by an electron-dense membrane, holding dispersed DNA strands and ribosome granules (A, inset). While some phytoplasmas were observed free floating in the SE lumen (A, B), others established a connection with the host cell at the plasma membrane or sieve element reticulum (SER) stack level (A, B). Plants responded to the infection with alterations at the ultrastructural level, such as callose accumulation at the sieve pore level (C, D), and SE protein filament condensation (arrows). SE protein filaments formed networks with different organization and density (B), that could plug pores at the sieve plates (C, inset). Five non-serial sections from 10 different plants were analysed. c, callose; CC, companion cell; m, mitochondrion; ph, phytoplasma; PPU, pore plasmodesma unit; SE, sieve element; ser, sieve element reticulum; sp, sieve plate. Scale bars: 1 µm.

In all infected lines, phytoplasmas were present exclusively in SEs (wild-type plants were chosen as representative and described in Fig. 11), with a disparate distribution over the SEs (data not shown, cf. Pagliari *et al.*, 2016). The phytoplasmas exhibited a well-preserved structure, with their typical pleomorphic profile, delimited by an electron-dense membrane, enclosing dispersed DNA strands and ribosome granules (Fig. 11). Pleomorphic phytoplasmas were observed to float freely in the SE lumen or to be linked to the SE plasma membrane or SER stacks (Fig. 11A, B). In all infected plant lines, SEs were characterized by various alterations, including a significant accumulation of protein filaments in their lumen (Fig. 11C, D). Protein filaments presented different organization patterns, from simple or branched strands to thin networks (Fig. 11A) or dense meshwork filling the SE lumen, surrounding phytoplasmas and plugging sieve plate pores (Fig. 11B, D).

**Fig. 12.**
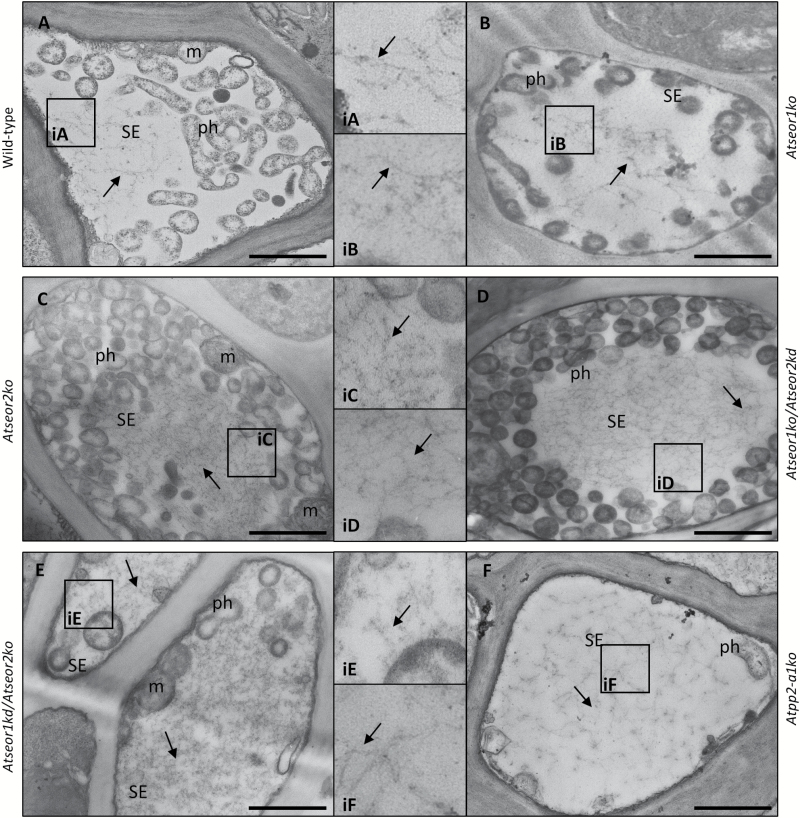
TEM micrographs of sieve elements in different infected *A. thaliana* lines. Different *A. thaliana* lines were indicated as follows: (A) wild-type, (B) *Atseor1ko*, (C) *Atseor2ko*, (D) *Atseor1ko/Atseor2kd*, (E) *Atseor1kd/Atseor2ko*, and (F) *Atpp2-a1ko*. In infected tissue, wild-type (A) and *Atpp2-a1ko* (F) SEs showed a massive presence of SE protein filaments (arrows, insets). Also in *A. thaliana* lines lacking one or both *AtSEOR* genes (B–E), infected SEs showed filaments with morphology and organization similar to those observed in wild-type plants (arrows, insets). For each condition, five non-serial sections from 10 different plants were analysed. CC, companion cell; m, mitochondrion; ph, phytoplasma; SE, sieve element. Scale bars: 1 µm.

In every infected section of Arabidopsis lines lacking one or both *AtSEOR* genes, SEs showed electron-dense filaments that could be straight, branched, or even organized into thin networks, with a morphology and organization similar to those observed in infected wild-type plants (Fig. 12B–E). Filaments were also observed in the infected SEs of *Atpp2-a1ko* plants (Fig. 12F).

### AtSEOR1, AtSEOR2, and AtPP2-A1 *gene expression analyses*

**Fig. 13.**
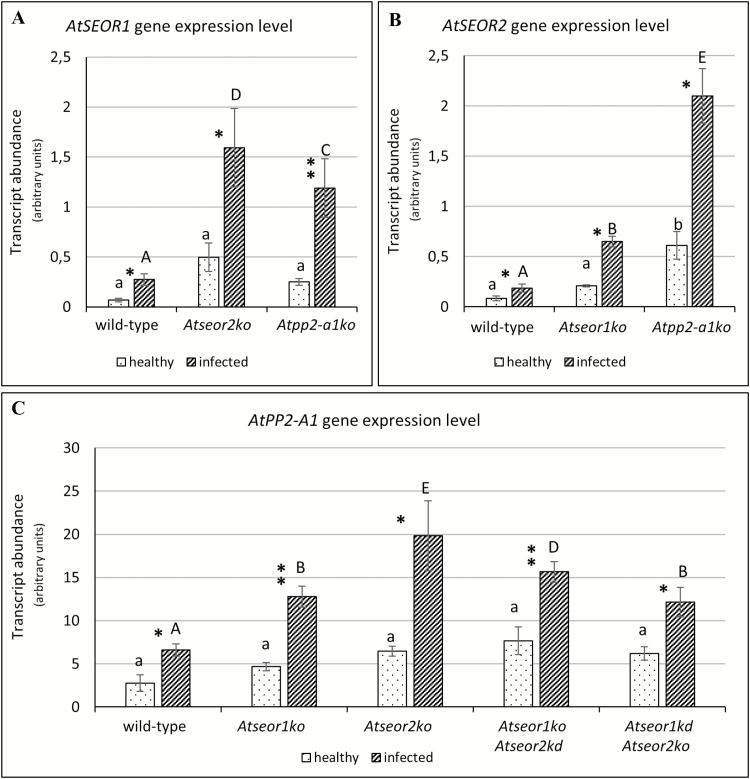
Gene expression level of healthy and infected wild-type and mutant *A. thaliana* lines. The expression of *AtSEOR1* (A), *AtSEOR2* (B), and *AtPP2-A1* (C) genes was analysed in healthy and infected plants of both wild-type and mutant lines by real-time RT–PCR. Expression values were normalized to the *UBC9* transcript level, arbitrarily fixed at 100, then expressed as mean normalized expression ±SD (transcript abundance). Every gene was significantly up-regulated in infected plants compared with healthy plants. No significant difference in expression was detected in healthy plants, except for *AtSEOR2* in *Atpp2-a1ko* plants. In infected plants, each gene was up-regulated in the mutants compared with the wild type. For each condition, three technical repeats from five different plants were carried out. Differences between healthy and infected conditions in each line were calculated using unpaired *t*-test. Family-wise significance and confidence level: 0.05. *P<0.05, **P<0.01. For comparisons among the different lines, two-way ANOVA followed by a Dunnett’s test was used, with wild-type values as control. Family-wise significance and confidence level: 0.05. Different letters indicate different significance levels. Healthy plants: a, no significant difference from wild-type values; b, *P*<0.05. Infected plants: A, no significant difference from wild-type values; B, *P*<0.05; C, *P*<0.01, D, *P*<0.001, E, *P*<0.0001.

To check if *AtSEOR1*, *AtSEOR2*, and *AtPP2-A1*genes are involved in the infection response, their expression was analysed in healthy and infected plants by real-time reverse transcription–PCR (RT–PCR) experiments. For each condition, three technical repeats in at least five individuals were investigated. For all samples, gene expression analyses are presented in Fig. 13, where the mean normalized expression of each gene is plotted as the transcript abundance compared with the *UBC9* expression level (set at 100). The expression level of the *AtSEOR1* and *AtSEOR2* genes was low, with MNE values fluctuating from 0.07 to 1.59 and from 0.08 to 2.10, respectively. *AtPP2-A1* presented a higher gene transcription level as compared with the *AtSEOR* genes, ranging from 2.77 to 19.84.

A compensatory general pattern was visible in the expression of SE filament genes in the different mutants. The expression levels of the other genes in question were a few times higher in the mutants than in wild-type plants. For instance, *AtSEOR1* expression was five times higher in healthy specimens of the *Atseor2ko* line and three times higher in the *Atpp2-a1ko* line than in healthy wild-type plants (Fig. 13A). In infected plants, *AtSEOR1* was overexpressed as compared with healthy plants by a factor of 3 in the wild-type, in *Atseor2ko* by a factor of 16, and in *Atpp2-a1ko* by a factor of 12. Similar tendencies, though not always significant, were recognizable in the other approaches (Fig. 13B, C).The unpaired *t*-test on MNE values showed that each gene analysed was significantly up-regulated in infected plants (Fig. 13). To compare the expression level of each single gene in every Arabidopsis line, a two-way ANOVA and a Dunnett’s test as post-hoc test were performed using the wild-type MNE values as negative controls. No significant differences were detected among expression levels in healthy plants, except for *AtSEOR2*, which was significantly up-regulated in healthy *Atpp2-a1ko* plants. On the other hand, in infected plants, each gene showed a significant increase in transcription levels in the mutant lines in comparison with the wild type (Fig. 13).

### Phytoplasma titre determination

**Fig. 14.**
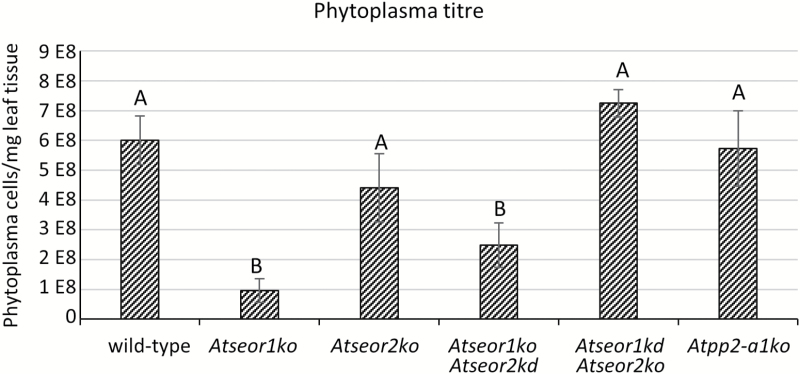
Phytoplasma titre in infected leaves of various sieve element protein *A. thaliana* mutants. Phytoplasma quantification in the different *A. thaliana* lines was performed by qPCR and expressed as the number of CY phytoplasma genome units (GUs) per 1 mg of leaf sample. The results are expressed as the mean ±SD. Wild-type, *Atseor2ko*, *Atseor1ko/Atseor2k*d, and *Atpp2-a1ko* plants were characterized by similar phytoplasma titres, while *Atseor1ko* and *Atseor1ko/Atseor2kd* plants showed lower mean phytoplasma concentrations. In every line, three technical repeats from 10 different plants were carried out. One-way ANOVA followed by a Dunnett’s test was used to determine significance, with wild-type values as control. Family-wise significance and confidence level: 0.05. Different letters indicate different significance levels: A, no significant difference from wild-type values; B, *P*<0.0001.

To correlate phloem impairment with pathogen replication capability and spread, phytoplasma titre was quantified in 10 infected plants from each line, performing three technical replicates every time. Molecular analyses confirmed the presence of phytoplasmas in every symptomatic plant. Wild-type, *Atseor2ko*, *Atseor1ko/Atseor2kd*, and *Atpp2-a1ko* plants had phytoplasma titres ranging from 95.55E+06 to 724.68E+06 phytoplasma GUs in 1 mg of leaf sample. One-way ANOVA and a Dunnett’s post-hoc test revealed significantly lower phytoplasma concentrations in *Atseor1ko* and *Atseor1ko/Atseor2kd* plants (95.55E+06 and 248.61E+06 phytoplasma GUs mg^–1^ of leaf sample, respectively) than in wild-type plants (600.87E+06 phytoplasma GUs mg^–1^ of leaf sample) (Fig. 14).

## Discussion

### *Phytoplasma infection induces phloem protein filament formation in* AtSEOR *mutant lines*

In Arabidopsis two non-redundant *SEO* genes, *AtSEOR1* and *AtSEOR2* (Rüping *et al.*, 2010; Anstead *et al.*, 2012), are regarded to be necessary for SE protein filament formation (Anstead *et al.*, 2012). In healthy plants, TEM pictures reveal the presence of SE protein filaments only in wild-type and *Atpp2-a1ko* plants (Figs 9, 10), in line with previous results (Anstead *et al.*, 2012). In contrast, infected plants of every line show a significant presence of SE protein filaments (Fig. 12). These findings led to the hypothesis that SE filaments are involved in plant response to pathogen attack and, under stress conditions, their formation becomes independent of the presence of AtSEOR1 or AtSEOR2. The effective mechanism that could regulate this phenomenon remains unknown and may involve those SE proteins that form the SE filament but are still unknown (Anstead *et al.*, 2012; Jekat *et al.*, 2013). Moreover, filament presence is accompanied by an up-regulation of SE protein genes in infected mutant lines in comparison with wild-type lines (Fig. 13). This phenomenon may indicate that, as already observed in other systems (Schneider *et al.*, 2002; Gierth and Mäser, 2007; Kawakatsu *et al.*, 2009; Ramundo *et al.*, 2014), plants try, in stressful conditions, to compensate in the mutants the lack of one protein by the enhanced production of the others.

### Wild-type SE protein filaments restrict phloem flow

The question now arises of whether occlusion by protein filaments restricts mass flow. Phytoplasma-infected plants show stunted growth and a diversity of alterations such as yellowing, leaf size reduction, and vein enlargement (Fig. 1). Such symptoms have commonly been attributed to impairment of phloem activities following phytoplasma infection (Braun and Sinclair, 1978; Kartte and Seemüller, 1991; Lepka *et al.*, 1999; Maust *et al.*, 2003). However, yellowing could also be due to down-regulation of photosynthetic genes (Hren *et al.*, 2009; Ji *et al.*, 2009; Taheri *et al.*, 2011), accompanied by a reduction of total chlorophyll content (Bertamini *et al.*, 2002*a*, *b*; Junqueira *et al.*, 2004; Zafari *et al.*, 2012).

The identical sieve tube conductance for CF in healthy wild-type and mutant lines (Fig. 7, 1–3) is in keeping with the similar mass flow rates found in wild-type and *AtSEOR1* mutants in earlier studies (Froelich *et al.*, 2011). On the other hand, the mass flow rates between infected wild-type and mutant plants differed considerably (Figs 7, 8), although midrib histology (Figs 3, 4) and phloem thickness (Fig. 5) show apparently identical modifications in response to infection in the different Arabidopsis lines. These observations indicate that wild-type plants are able to occlude the sieve pores, while the mutants are unable to do so. Apparent occlusion by filamentous proteins in wild-type Arabidopsis plants contrasts with some earlier observations (Froelich *et al.*, 2011), but it is in agreement with others (Ernst *et al.*, 2012; Jekat *et al.*, 2013). The reason for the contradictory results may lie in the fact that SE proteins do not aggregate in intact wild-type plants, but their presence is massive in plants stressed by phytopathogens (Musetti *et al.*, 2013) or by wounding (Ernst *et al.*, 2012; Jekat *et al.*, 2013). Both stress reactions may rely on Ca^2+^ influx, mediated by stress-activated Ca^2+^-permeable channels (Hafke *et al.*, 2009; van Bel *et al.*, 2014), which would confer coagulation of SEOR proteins.

Lack of one of the SEOR proteins may explain the inability to seal sieve tubes in infected mutants (Figs 7, 4–6) despite the phytoplasma-imposed stress. Masses of SE protein filaments showing up in TEM pictures of phytoplasma-infected mutant plants (Fig. 12) may result from compensatory expression of other genes (Fig. 13). As in forisomes, a structural arrangement of filamentous SEOR proteins may provide the presumptive spatial conditions for Ca^2+^ binding to result in plug formation. If one of the proteins is lacking, effective occlusion structures may not be formed.

### Phloem impairment does not effectively affect pathogen spread

Phytoplasma-triggered phloem impairment has been postulated to be a plant strategy to limit pathogen spread (Lherminier *et al.*, 2003; Gamalero *et al.*, 2010; Musetti *et al.*, 2013). Remarkably, the fact that widely different phytoplasma titres do not concur with the phloem mass flow rates in the respective Arabidopsis lines (Figs 7, 14) suggests an alternative defence mechanism for pathogen containment or control. This conclusion is compatible with the view that phytoplasma spread is not only dependent on phloem mass flow (Garcia-Chapa *et al.*, 2003; Wei *et al.*, 2004; Buxa *et al.*, 2015). The low phytoplasma titres found in *Atseor1ko* and *Atseor1ko/Atseor2kd* plants and the putative involvement of AtSEOR2 protein in plant immune signalling (Afzal *et al.*, 2013) hints at a connection between SE protein and the plant immunity system, for which further experiments are envisaged.

### Conclusions

The prime goal of this study was to assess a relationship between phytoplasma infection and SE filament agglutination. In infected plants, SE protein filament formation can overcome the absence of *AtSEOR1* or *AtSEOR2*, which is supportive of speculations regarding the engagement of other proteins in the formation of SE filaments (Anstead *et al.*, 2012; Jekat *et al.*, 2013). Phloem flow analysis in healthy and infected plants indicates that SE protein filaments impair phloem transport in infected wild-type but not in infected mutant plants. Absence of a correlation between phloem impairment and pathogen multiplication indicates that limitation of mass phloem is not an effective strategy to combat phytoplasma spread. Perhaps SEOR proteins are engaged in other defence mechanisms.

## Abbreviations:

CF: carboxyfluorescein
CFDA: 5,6-carboxyfluorescein diacetate
CLSM: confocal laser scanning microscopy
CY: Chrysanthemum yellows
NASC: Nottingham Arabidopsis Stock Centre
PP2: phloem protein 2
SE: sieve element
SEO: sieve element occlusion
SEOR: sieve element occlusion related
SER: sieve endoplasmic reticulum.
